# Pharmacological Preconditioning Improves the Viability and Proangiogenic Paracrine Function of Hydrogel-Encapsulated Mesenchymal Stromal Cells

**DOI:** 10.1155/2021/6663467

**Published:** 2021-07-28

**Authors:** Francesco K. Touani, Melanie Borie, Feryel Azzi, Dominique Trudel, Nicolas Noiseux, Shant Der Sarkissian, Sophie Lerouge

**Affiliations:** ^1^Centre de Recherche du Centre Hospitalier de l'Université de Montréal (CRCHUM), Montreal, QC, Canada; ^2^Department of Pharmacology and Physiology, Université de Montréal, QC, Montreal, Canada; ^3^Institut du Cancer de Montréal (ICM), QC, Canada; ^4^Department of Pathology and Cellular Biology, Université de Montréal, Montreal, Canada; ^5^Department of Surgery, Université de Montréal, Montreal, QC, Canada; ^6^Department of Mechanical Engineering, École de Technologie Supérieure (ÉTS), Montreal, QC, Canada

## Abstract

The efficacy of cell therapy is limited by low retention and survival of transplanted cells in the target tissues. In this work, we hypothesize that pharmacological preconditioning with celastrol, a natural potent antioxidant, could improve the viability and functions of mesenchymal stromal cells (MSC) encapsulated within an injectable scaffold. Bone marrow MSCs from rat (rMSC) and human (hMSC) origin were preconditioned for 1 hour with celastrol 1 *μ*M or vehicle (DMSO 0.1% *v*/v), then encapsulated within a chitosan-based thermosensitive hydrogel. Cell viability was compared by alamarBlue and live/dead assay. Paracrine function was studied first by quantifying the proangiogenic growth factors released, followed by assessing scratched HUVEC culture wound closure velocity and proliferation of HUVEC when cocultured with encapsulated hMSC. In vivo, the proangiogenic activity was studied by evaluating the neovessel density around the subcutaneously injected hydrogel after one week in rats. Preconditioning strongly enhanced the viability of rMSC and hMSC compared to vehicle-treated cells, with 90% and 75% survival versus 36% and 58% survival, respectively, after 7 days in complete media and 80% versus 64% survival for hMSC after 4 days in low serum media (*p* < 0.05). Celastrol-treated cells increased quantities of proangiogenic cytokines compared to vehicle-pretreated cells, with a significant 3.0-fold and 1.8-fold increase of VEGFa and SDF-1*α*, respectively (*p* < 0.05). The enhanced paracrine function of preconditioned MSC was demonstrated by accelerated growth and wound closure velocity of injured HUVEC monolayer (*p* < 0.05) in vitro. Moreover, celastrol-treated cells, but not vehicle-treated cells, led to a significant increase of neovessel density in the peri-implant region after one week in vivo compared to the control (blank hydrogel). These results suggest that combining cell pretreatment with celastrol and encapsulation in hydrogel could potentiate MSC therapy for many diseases, benefiting particularly ischemic diseases.

## 1. Introduction

Cardiovascular disease (CVD) is a leading cause of mortality, and of these deaths, 85% are due to ischemic events [1, 2]. Clinical management includes fibrinolytic therapy, primary percutaneous coronary intervention, or bypass graft surgery to restore blood flow [3–7]. However, these interventions cannot regenerate dead cells and scar tissues.

Mesenchymal stromal cell (MSC) therapy is a promising treatment for various degenerative diseases by triggering neovascularization and stimulating tissue regeneration [8–13]. MSCs, which are abundant and only weakly immunogenic, are readily used in several clinical trials in regenerative medicine for the treatment of ischemic diseases such as hind limb ischemia [14, 15] and ischemic heart diseases [8, 16]. These cells are able to secrete proangiogenic, chemoattractant, and antiapoptotic mediators useful for the recovery of ischemic tissue [17–19]. However, the efficacy of cell therapy remains limited due to the poor retention, diminished survival, and poor functionality of cells, especially when transplanted in such ischemic, inflammatory, and oxidative microenvironments [16, 17]. In this context, injectable scaffolds have been proposed to localize, anchor, and protect cells in the target tissues [20–24]. Our laboratory has developed scaffolds composed of thermosensitive hydrogels based on chitosan, a natural biocompatible and biodegradable polymer obtained by deacetylation of chitin [25]. Particularities of our hydrogels include physiological pH and low viscosity at room temperature which enables easy cell loading and injectability through small needles and rapid gelation at body temperature with desirable mechanical properties and cytocompatibility [26, 27]. However, the hydrogel does not prevent and may even exacerbate the lack of oxygen and nutrients reaching the cells, which can negatively affect their viability and therapeutic effects. We therefore propose to combine encapsulation with cell preconditioning to improve cell retention, survival, and function.

Preconditioning consists in activating cytoprotective pathways by either exposing cells to a sublethal environment [28, 29], by transfection of cell survival genes [30, 31], or by conditioning cells with pharmacological molecules to activate functional and protective cellular pathways [32–36]. Pharmacological preconditioning with certain natural classes of antioxidants could be particularly interesting. Celastrol is a natural potent antioxidant extracted from the bark of the roots of Tripterygium wilfordii plant used in traditional oriental medicine for many pathologies such as autoimmune inflammation [37] and chronic diseases [38]. Our team showed that a short burst treatment with celastrol protects cells against hypoxia and oxidative damage as found in ischemic tissues and increases cell paracrine competence with the enhanced expression and secretion of many potent bioactive factors [33, 34, 39].

In this study, we demonstrate that combining both cell encapsulation and pharmacological preconditioning enhances the viability and the proangiogenic paracrine function of MSC in vitro and in vivo and could be used to improve the outcomes of cell therapy for ischemic diseases.

## 2. Materials and Methods

### 2.1. Hydrogel Preparation

The chitosan thermosensitive hydrogel is prepared by mixing an acidic solution of chitosan and a gelling agent solution [26], which were prepared as follows (Figure S1). Chitosan (Kitomer, PSN 326-501, Premium Quality, Mw 250 kDa, DDA 94%; Marinard Biotech) was purified [26] and solubilized at an initial concentration of 3.33% (*m*/*v*) in 0.1 N hydrochloric acid for 3 hours at 500-700 rpm (Heidolph RZR 2021). The chitosan solution was then autoclaved at 121°C for 20 min and stored at 4°C until experiments.

The gelling agent (GA) solution is a combination of two weak bases, namely, sodium hydrogen carbonate (SHC, Solon, OH, USA) and phosphate buffer (PB) [26]. PB solution was prepared at 0.2 M and pH 8 by mixing sodium phosphate dibasic (SDP, Sigma-Aldrich, ON, CA) and monobasic (SPM, Sigma-Aldrich, ON, CA) with volume ratio 0.932/0.068 in Milli-Q water. Then, SHC was solubilized in a PB solution and vortexed until complete solubilization. The GA solution (PB 0.2 M-SHC 0.375 M) was then sterilized by filtration through a 0.22 *μ*m filter (Corning incorporated, NY, USA) and stored at 4°C until experiments.

### 2.2. Cell Culture

The isolated bone marrow-derived rMSCs from male Sprague Dawley [40] (Charles River, QC, Canada) were cultured with alpha minimum essential medium (MEM, Gibco, USA) supplemented with 10% Fetal Bovine Serum (FBS, Gibco, USA), while bone marrow-derived hMSCs (Lonza Inc., ON, Canada) were cultured with NutriStem XF (Biological Industries, Israel) supplemented with 0.6% MSC NutriStem XF Suppl. Cells were seeded at 6,600/cm^2^ density and cultured up to 90% confluence before experiments. Human umbilical vein endothelial cells (HUVEC: ATCC, ON, CA) were cultured with endothelial growth medium 200 (EGM) and 5% FBS, supplemented with 2% low serum growth supplement (LSGS, Life Technologies) at 9,000/cm^2^ density. Before seeding of HUVEC, dishes were coated with porcine gelatin-type A (Sigma-Aldrich, Oakville, ON, Canada) diluted in sterile phosphate buffer saline (PBS1X, Sigma-Aldrich) for 2 hours. All experiments were carried out only with alpha MEM. Cells were used within passages 2 and 8 for experiments.

### 2.3. Pharmacological Preconditioning of Cells

Cell preconditioning, also called pharmacooptimisation [34] was carried out with celastrol (Cayman Chemical, Ann Arbor, MI, USA), according to the previously described method [39]. Briefly, adhered cells (at 90% of the confluence) were stimulated for 1 hour with celastrol 1 *μ*M (dissolved in DMSO 0.1% *v*/*v*) or vehicle (DMSO 0.1% (*v*/*v*)) at 37°C, 5% CO_2_ in alpha MEM 1% FBS. This concentration of celastrol was chosen following preliminary optimization assay performed on nonencapsulated cells coated by a 3 mm layer of hydrogel, as described in supplemental data. Cells were rinsed three times with alpha MEM 1% FBS and left to recover for 4 hours at 37°C and 5% CO_2_ in alpha MEM 10% FBS, before encapsulation in the hydrogel.

### 2.4. Preparation of MSC-Loaded Hydrogel

Hydrogel preparation for cell encapsulation consisted of mixing a CH solution, GA solution, and cell suspension at the volume ratio 0.6, 0.2, and 0.2, respectively. The mixing was made in 2 consecutive steps: first, mixing was done by 15 consecutive plunger shuffles of syringes from CH and GA solutions [26, 41]. The preformed gel (still liquid at room temperature) was immediately mixed 15 times with the cell suspension. The final composition of the hydrogel was CH 2% (*w*/*v*)-SHC 0.075 M-PB 0.04 M.

For in vitro studies, a volume of 200 *μ*L of hydrogel (containing 7 × 10^5^ cells) was deposited in 48-well plates and left to gel for 3 minutes at 37°C, 5% CO_2_. Then, 500 *μ*L of complete alpha MEM was added on the top of the gel and the plates were further incubated at 37°C. The alpha MEM was renewed on day 4. As described below, cell viability and paracrine factor release were first studied. The proangiogenic properties were then directly assessed using a wounded HUVEC monolayer assay (“scratch test”) and coculture of HUVEC with encapsulated hMSC. All results were normalized to the vehicle-treated cells.

### 2.5. In Vitro Cell Viability

Cell viability was measured on days 1 and 7 on cells loaded in hydrogel (7 × 10^5^/200 *μ*L of the hydrogel) and incubated in complete culture media (rMSC, hMSC). Cell viability in low serum culture media (alpha MEM 0.2% FBS) was also assessed, after 24 h of encapsulation as described above. For this, culture media were changed to low serum media culture (500 *μ*L of alpha MEM 0.2% FBS) and cells were further incubated at 37°C, 5%CO_2_, for 3 days. All results were normalized to the vehicle-treated cells as 100%. Cell viability was first quantified by measuring the metabolic activity using alamarBlue assay (Biotium Inc., Fremont, CA, USA). Resazurin (10% (*v*/*v*)) was diluted in complete medium and incubated for 3 h before measurement of fluorescence emission at 560-590 nm (BioTek Instruments Inc., Synergy 4, USA). Viability was also confirmed with live/dead assay (Life Technologies, ON, CA). Cells were incubated with serum-free alpha MEM containing 2 *μ*M calcein AM and 5.5 *μ*M homodimer ethidium at 37°C, 5% CO_2_, for 45 minutes. Pictures were taken with a fluorescence inverted microscope (Leica DMIRB) at 50x magnification.

### 2.6. Paracrine Activity Assessment of Hydrogel-Encapsulated Cells

To assess paracrine release, conditioned media were obtained by incubating the hydrogel loaded with cells in low serum culture media (alpha MEM 0.2% FBS). For this, culture media were changed to low serum medium culture (500 *μ*L of alpha MEM 0.2% FBS) and the cell-loaded hydrogel was further incubated at 37°C, 5% CO_2_, for 3 days. Paracrine activity assessment was carried out with ProcartaPlex multiplex immunoassay (ThermoFisher Scientific Inc., MA, USA) by measuring quantitatively proangiogenic protein concentrations (VEGFa, FGF2, and SDF-1*α*) in the conditioned media. Cell viability in low serum media was also assessed, as described above.

### 2.7. Scratch Test or Wound Healing Assessment

**Figure 1:**
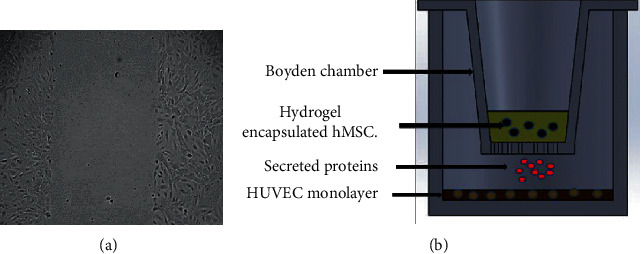
(a) Initial wound of HUVEC monolayer made by the Woundmaker. (b) Schematic representation of the coculture of HUVEC with hydrogel-encapsulated hMSC seeded in a Boyden chamber. Both cells were incubated in alpha MEM 0.2% FBS.

To perform the scratch test, adhered HUVEC (15,000/well) were cultured overnight in 96-well plates (Essen BioScience, Inc.) previously coated with gelatin 1% for 2 hours. The next day, the HUVEC monolayer was scratched with a Woundmaker™ (Figure 1(a); Essen BioScience, Inc.). Plates were washed with PBS 1X. Conditioned media (alpha MEM0.2% FBS) from hydrogel-loaded MSC were centrifuged for 15 min at 1,300 rpm (to remove particles of hydrogel) and diluted at 50% (with a fresh alpha MEM 0.2% FBS). A volume of 50 *μ*L of conditioned media was added in each well. Plates were incubated at 37°C, 5%CO_2_, and images were taken every 2 hours for 24 hours with IncuCyte™ ZOOM software (Essen BioScience, Inc.). The rate of wound closure of HUVEC incubated in conditioned media from celastrol-pretreated hMSC was compared with conditioned media from vehicle-pretreated cells. Complete EGM200 was used as positive control while alpha MEM 0.2% served as negative control.

### 2.8. Coculture of HUVEC with Encapsulated hMSC

Coculture of HUVEC with hMSC-loaded hydrogel was performed using Boyden chambers (Figure 1(b), Corning Inc., NY, USA). HUVEC (15,000/well; approximately 60% of the confluence) were left to adhere overnight in gelatin-coated 24-well plates. The next day, hMSCs were treated with celastrol or vehicle and incorporated into the hydrogel solution (as described above). A volume of 200 *μ*L of hydrogel was introduced within Boyden chambers and left to gel for 5 minutes at 37°C. The Boyden chambers were then transferred into the 24-well plates, and 2 mL of alpha MEM 0.2% FBS was added before further incubation at 37°C, 5%CO_2_. HUVEC growth was evaluated by comparing cell metabolic activity at 0 h, 24 h, and 48 h using resazurin. HUVEC incubated with complete EGM and alpha MEM 0.2% (in the presence of hydrogel without cells as hydrogel blanks) were used as positive and negative controls, respectively.

### 2.9. In Vivo Paracrine Functions

A pilot in vivo study was carried out in compliance with guidelines from the Institutional Animal Protection Committee of the CRCHUM. First, rMSCs were preconditioned as described in Section 2.3. Then, cells were trypsinated and stained with Vybrant™ DID (1,1′-dioctadecyl-3,3,3,3′-tetramethyl indodicarbocyanine-4-chlorobenzene sulfonate salt) cell-labeling solution for 20 min in serum-free media according to the manufacturer protocol (Life Technologies). Cells preconditioned by celastrol or vehicle were incorporated into the hydrogel solution as previously described (3 × 10^6^/200 *μ*L of hydrogel), and both solutions were subcutaneously injected in the dorsum of 8 female Sprague Dawley rats (Charles Rivers) using a 23 G needle. A third injection consisted in the hydrogel without cells (control group). Labeled cell in vivo signal was measured on days 0 and 7 by fluorescence imaging (eXplore Optix™ MX2 system, ART Advanced Research Technologies, Inc., Canada). The region of interest (ROI) was scanned using an excitation source at 670 nm with a spot size of 1.0 mm.

On day 7, rats were euthanized, and gels were immediately excised, fixed in 10% formalin, and embedded in paraffin. Histological sections of 6 *μ*m thickness were fixed on charged glass slides. Immunohistochemical staining for von Willebrand factor (vWF), and CD68 was carried out on paraffin-embedded formalin-fixed samples using the automated Bond RX staining platform from Leica (Biosystems, Australia). Sections were deparaffinised inside the immunostainer. Antigen recovery was conducted using the specific proprietary solution from Leica Biosystems: Heat-induced Epitope Retrieval (ER) with ER1 low-pH buffer for CD68 or ER2 high-pH buffer for vWF as long as 20 minutes. Sections were then incubated with 150 *μ*Lof each primary antibody, VWF (MilliporeAB7356; 1-200) and CD68 (Abcam AB31630; 1-300), for 15 min at room temperature. Detection of specific signal was acquired by using Bond Polymer DAB Refine kit (#DS9800, Leica Biosystems) according to providers' recommendations. Slides were counterstained automatically with hematoxylin included in the detection kit. The thickness of the macrophage-rich layer (CD68+) was blindly measured at 3 different locations in the inflammatory area around the gels (large, medium, and small) with NDPview software, and an average was calculated. The neovessel density (vWF+ intensity) in the granulation tissue at the perigel area was scored by a pathologist (FA) who was blinded to experimental conditions. Scale spanned from + to +++ and was then classified into 2 different groups. Samples scored as +++ were categorized as high response and samples scored below +++ (+ and ++) were categorized as low response.

### 2.10. Statistical Analysis

Each experiment was performed at least in triplicate. *N* (number of repeated experiments) and *n* (total sample number) are indicated in each figure caption. All results are expressed in the *mean* ± *standard* *error* of the mean (SEM). *T*-test and ANOVA (Statgraphics XVII) were used to determine the statistical difference between groups, with *p* values below 0.05 considered statistically significant. The fisher (*F*) and Kolmogorov-Smirnov test were used to compare the variances between groups and the normality of the analyzed data, respectively. Student *T*-test analyses were performed on data with a normal distribution, whereas a nonparametric Mann-Whitney test was performed on data that did not follow a normal distribution. Chi square (EZ SPSS) was used to analyze the scores of neovessel density between samples.

## 3. Results

### 3.1. Effect of Pharmacological Preconditioning on the Viability of Hydrogel-Encapsulated MSC

**Figure 2:**
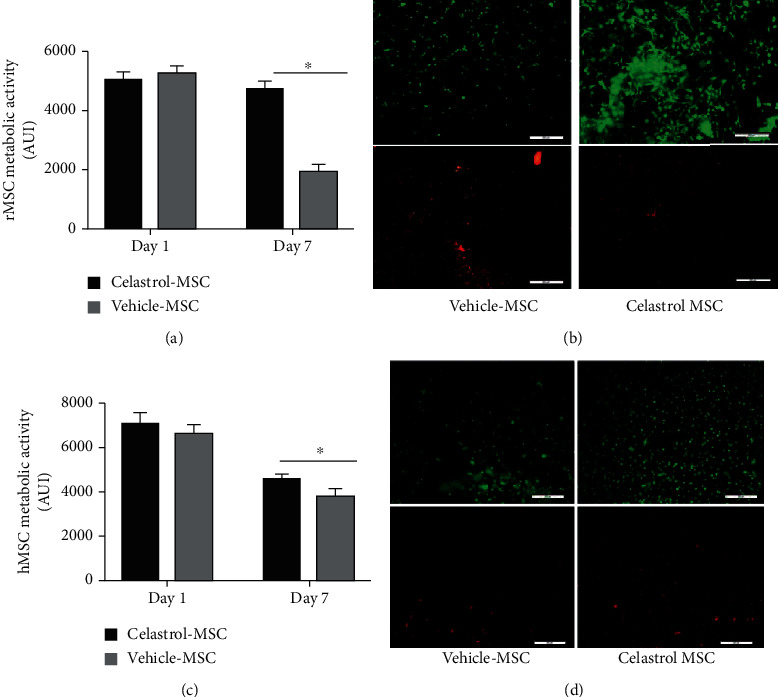
Celastrol increases viability of encapsulated MSC in normal medium: (a) metabolic activity at days 1 and 7 of rat MSC preconditioned with celastrol 1 *μ*M or vehicle (DMSO 0.1% *v*/*v*), mean ± SEM; b) live/dead pictures taken at day 7; *n* = 8, *N* = 3; (c) metabolic activity at days 1 and 7 of human MSC preconditioned with celastrol 1 *μ*M or vehicle, mean ± SEM, *n* = 14, *N* = 5; (d) live/dead pictures taken at day 7 (viable cells: green, dead cells: red, scale bar 200 *μ*m); ^∗^*p* < 0.05 versus the vehicle at the same time point.

The preliminary proof of concept was performed with MSC (hMSC and rMSC) cultured in 2D and covered by a 3 mm thick hydrogel layer, which limits nutrient diffusion. Preconditioning with celastrol enhanced hMSC viability in a dose-dependent manner (see Figures 2S and 3S in supplemental data). A single dose (1 *μ*M) was then chosen for further studies with cells encapsulated in 3D in the hydrogel. The efficacy of cell preconditioning was evaluated in complete and in low serum medium, first with rMSCs which are readily available and useful for preclinical studies. Results were then confirmed with hMSC, since these are used for in vivo tests.

Figure 2(a) presents the metabolic activity of rMSC, treated with celastrol 1 *μ*M or the vehicle, encapsulated in the hydrogel, and incubated in complete medium (10% FBS), reported as the percentage of the fluorescence signal measured for the vehicle group at day 1. While there was no significant difference between the two groups at day 1, the metabolic activity after 7 days was significantly higher for the celastrol-treated cells, with about 90% versus 36% of the initial value of the vehicle-treated cells at day 1 (Figure 2(a), *p* < 0.05). This suggests that pharmacological preconditioning with celastrol 1 *μ*M had a strong effect on maintaining viability of encapsulated rMSC. This was confirmed by live/dead assay at day 7 (Figure 2(b)). Results were confirmed in hMSC with a maintenance of their metabolic activity at 75% versus 58% for celastrol- and vehicle-treated cells, respectively, on day 7 (Figure 2(c), *p* < 0.05). Live/dead assay confirmed these results (Figure 2(d)).

**Figure 3:**
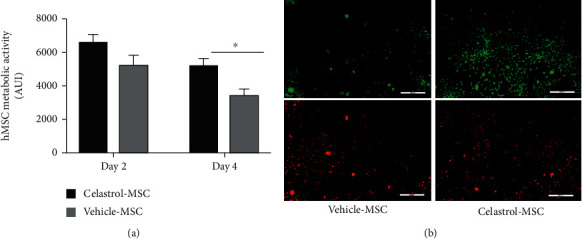
Celastrol increases the viability of encapsulated MSC in low serum media: (a) metabolic activity, at days 2 and 4, of hMSC pretreated with 1 *μ*M of celastrol or vehicle (DMSO 0.1% *v*/*v*), encapsulated in 200 *μ*L of hydrogel, and incubated in low serum media; mean ± SEM, *n* = 7, *N* = 3 (^∗^*p* < 0.05 versus the vehicle at each time point); (b) live/dead pictures taken at day 4 (viable cells: green, dead cells: red; scale bar = 200 *μ*m).

To verify the benefits of pharmacooptimization in hydrogel-encapsulated hMSC placed in a nutrient-deficient environment, cells were incubated in low serum media (0.2% FBS). Cell metabolic activity was then assessed at days 2 and 4. The metabolic activity of cells preconditioned with celastrol 1 *μ*M was increased by 27% and 56% compared to the vehicle-treated hMSC at days 2 and 4, respectively (Figure 3(a), *p* < 0.05). Moreover, the comparison of cell metabolic activity at days 2 and 4 showed that about 80% of celastrol-treated cells remained viable on day 4, compared to 64% for the vehicle-treated cells. Live/dead staining confirmed these results, showing a clear increase in the number of live cells in the celastrol group at day 4 (Figure 3(b)).

**Figure 4:**
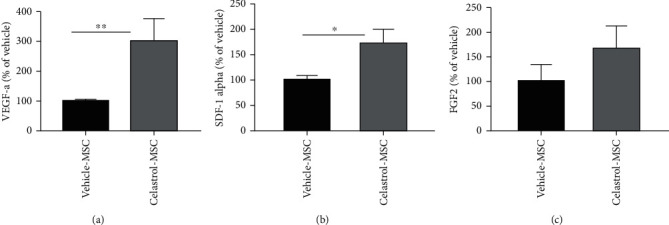
Celastrol preconditioning increases the amount of angiogenic factors released by encapsulated hMSC: (a) VEGF-a (mean ± SEM, *n* ≥ 6, *N* = 4); (b) SDF-1*α* (mean ± SEM, *n* ≥ 12, *N* = 4); and (c) FGF-2 (mean ± SEM, *n* = 6, *N* = 3) concentrations in conditioned media of hydrogel-encapsulated hMSC on day 4 (^∗∗^*p* < 0.01 and ^∗^*p* < 0.05; no significant difference between groups with FGF-2 (mean ± SEM, *n* = 6, *N* = 3).

Regeneration of damaged tissues by MSC therapy does require not only cell survival but also an efficient paracrine activity in target tissues. Particularly, in ischemic tissues, MSC paracrine activity is essential to promote neoangiogenesis and subsequent tissue reperfusion. Therefore, the conditioned media from hydrogel encapsulated hMSC in low serum were retrieved on day 4 and used to measure the concentration and bioactivity of 3 main cytokines involved in the revascularization, cell migration, and proliferation, namely, VEGFa, SDF-1*α*, and FGF-2 (Figure 4). The concentrations of VEGFa and SDF-1*α* were significantly increased for celastrol-pretreated hMSC, with a 3-fold (8885 ± 261 vs. 25479 ± 2331 pg/mL, *p* < 0.01) and 1.8-fold increase (1212 ± 116 vs. 2698 ± 276 pg/mL, *p* < 0.05) compared to the vehicle-treated cells, respectively. Despite a clear trend for its increase, the difference for FGF-2 content between the celastrol (68 ± 5.8 pg/mL) and vehicle- (41 ± 8.3 pg/mL) pretreated hMSC groups did not reach statistical significance (*p* = 0.26). Raw data of paracrine activities are found in supplemental data (Figures 4S).

These data suggest that celastrol-treated hMSCs encapsulated in the chitosan hydrogel have enhanced proangiogenic bioactivity compared to the vehicle-treated controls. The functional aspect of these results was validated in two in vitro assays, namely, scratch test experiments and HUVEC-hMSC coculture experiments.

### 3.2. Effect of Pharmacological Preconditioning on Paracrine Activities of Hydrogel-Encapsulated hMSC

**Figure 5:**
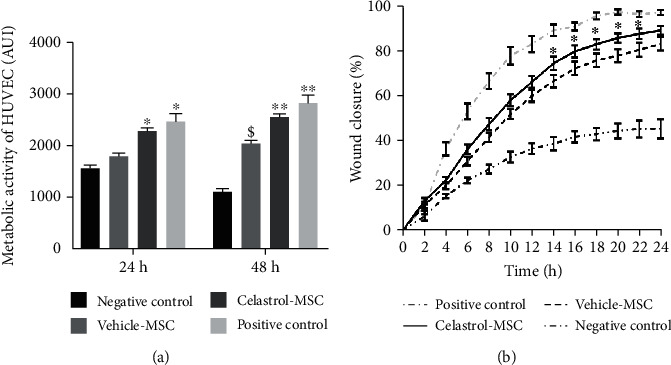
Celastrol preconditioning increases paracrine proangiogenic activity. (a) HUVEC growth when cocultured with hydrogel-encapsulated hMSC. Alpha MEM 0.2% FBS and EGM were used as negative and positive controls, respectively (mean ± SEM, *n* ≥ 3, *N* ≥ 2); ^∗^*p* < 0.05 versus the vehicle at 24 h; ^∗∗^*p* < 0.05 versus the vehicle at 48 h; ^$^*p* < 0.05 versus alpha MEM 0.2% FBS at 48 h. (b) Effect of paracrine activity of hydrogel-encapsulated hMSC on wound closure; mean ± SEM, *n* ≥ 9, *N* = 2; negative control: alpha MEM 0.2% FBS; positive control: EGM (^∗^*p* < 0.05 versus the vehicle group at each time point).

Figure 5(a) presents the growth of HUVEC when cocultured with hydrogel-encapsulated hMSC, preconditioned with celastrol or vehicle. Paracrine activity of hMSC had a clear effect on HUVEC as shown by increased HUVEC growth compared to the negative control (HUVEC in alpha MEM 0.2% FBS) (*p* < 0.05, Figure 5(a)). This effect was further strengthened by celastrol preconditioning, with 28% and 26% increase compared to the vehicle-treated cells after 24 and 48 h respectively (*p* < 0.05), and cell growth reached levels similar to the positive control group of cells grown in complete media supplemented with serum and endothelial growth factors (EGM), while a significant difference between vehicle-treated cells and the positive control group was observed at 24 h and 48 h (*p* < 0.05).

In the next experiment, a wound created on HUVEC monolayer was incubated with conditioned media from celastrol- or vehicle-treated hMSC in order to assess the velocity of wound closure. Alpha MEM 0.2% FBS and EGM were used as negative and positive controls, respectively. Both conditioned media significantly increased wound closure compared to alpha MEM (Figure 5(b), *p* < 0.05). However, wound closure in the celastrol group was accelerated compared to the vehicle group at each time point, and the difference reached statistical significance for the time points between 14 h and 22 h (*p* < 0.05).

### 3.3. Paracrine Function Assessment In Vivo

**Figure 6:**
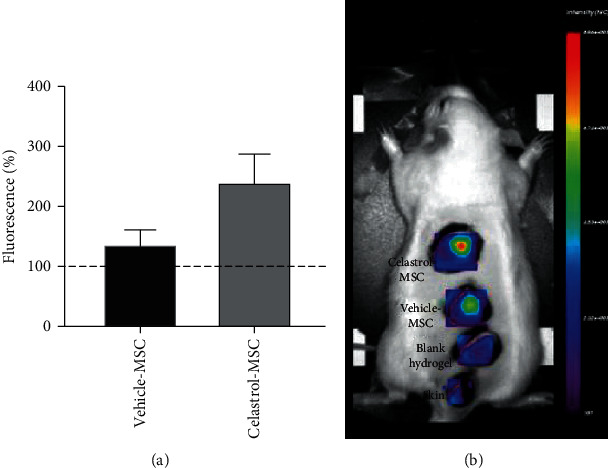
(a) Celastrol-treated hydrogel-encapsulated Vybrant-stained MSCs display 2.4-fold higher retention compared to vehicle-treated MSC at day 7 (mean ± SEM, *n* = 8, *N* = 2). (b) Representative fluorescence imaging of Vybrant-stained encapsulated MSC at 7 days; blank: hydrogel alone containing no cells; skin: no hydrogel or cells.

After injection of hydrogel-loaded cells in rats, the fluorescence intensity emitted by the encapsulated, Vybrant-stained cells was measured immediately following injections and after one week. Fluorescence was expressed in percent of day 0 (Figure 6). From day 0 to day 7, the signal emitting from the celastrol-treated cell group is higher than that from the vehicle-treated cell group, with a 2.4- and 1.4-fold increase respectively on day 7, without however reaching statistical significance.

Histology showed that at 7 days, chitosan gel was surrounded by inflammatory infiltrate mainly containing immune cells (polynuclear cells, lymphocytes) and granulation tissue.

**Figure 7:**
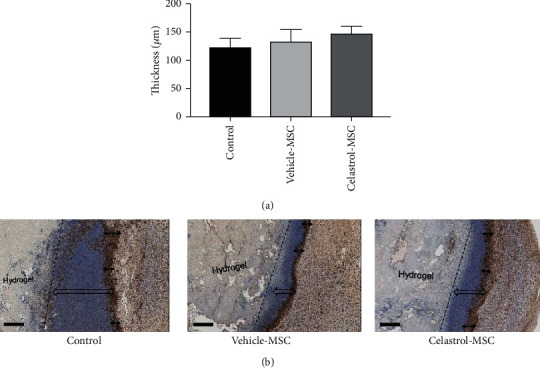
(a) Thickness of the macrophage-rich region around the implant on day 7. The control group was the hydrogel without cells. (b) Representative images showing macrophage infiltrations in the peri-implant region for each treatment group; arrows (↔) illustrate the thickness of the macrophage-rich region (brown, CD68 staining, scale bar 500 *μ*m), and arrows (⇔) illustrate the total immune cell infiltrations in the peri-implant region.

Figure 7 shows an immunomodulation effect of MSCs with a reduced area of inflammatory cell presence in the peri-implant region compared to the control group containing no cells. However, closer examination shows a trend of increasing macrophage infiltration in the peri-implant region by 8% and 21% with vehicle- and celastrol-pretreated MSCs, respectively, compared to the control condition (blank hydrogel).

**Figure 8:**
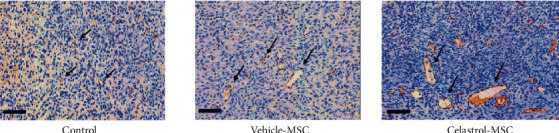
Representative images showing the increase of neovessel density with MSC in the peri-implant region (granulation tissue). Celastrol-treated MSCs display the highest neovessel density. Arrows indicate neovessels following vWF staining (brown); scale bar 100 *μ*m.

Table 1 presents the scores of neovessel density evaluated in the granulation tissue at the perigel area. The scores were divided into low- and high-response group. The score of neovessel density in the peri-implant region of celastrol-treated MSC was highest (7 cases out of 8) followed by the vehicle-treated MSC (5 cases out of 8) and only 1 case out of 6 in the control group (gel without cells). The difference between vehicle treatment and celastrol-preconditioned cells did not reach statistical significance; however, only the celastrol-pretreated hMSC group showed significantly higher neovessel density compared to the control group (*p* = 0.008).  Figure 8 shows an illustration of representative images of neovessel density in the peri-implant region. See the details of the scoring in the supplementary data (Table 1 S).

## 4. Discussion

Herein, we report that preconditioning MSC with celastrol for 1 hour prior to encapsulation within a chitosan hydrogel improves cell viability and proangiogenic paracrine activity as demonstrated through the increase of GF release, HUVEC proliferation, velocity of wound closure, and increase in neovessel number in the peri-implant region in vivo.

These results are in line with our previous work on cells without hydrogel, showing that a short burst treatment of cells with celastrol protects against hypoxia and oxidative stress-induced death [33, 39]. Protection was achieved by activation of survival kinases including pAKT and pERK and of heat shock and antioxidant response pathways with HO-1 and HSP70 protein expression [34, 39]. Such rapid (1 h) and simple in vitro pretreatment prior to cell transfer could enhance the efficacy of various forms of cell therapies for ischemic diseases such as hind limb ischemia.

Demonstration of the efficacy of celastrol on cells encapsulated in a 3D scaffold is interesting since scaffolds are increasingly used to enhance the outcome of cell therapy. While simple cell injection with saline is known to lead to rapid cell loss through both migration and death [20, 42, 43]. Combining cells with an injectable scaffold can enhance cell retention and survival and shield cells from immune attack [20, 21, 44]. The injectable thermosensitive chitosan hydrogel used in this study combines several advantages for cell therapy and tissue engineering applications. It is liquid at room temperature and rapidly gels at body temperature with desirable mechanical properties and cytocompatibility. It has shown very promising results in vitro as local delivery system of T lymphocytes for cancer immunotherapy [45] and for MSC [26].

However, encapsulated cells can still suffer from the lack of oxygen and nutrients or from oxidative stress in the target tissues. In addition, in this hydrogel, as in most scaffolds, cell survival can be impaired by several factors such as mechanical stress during the encapsulation process, reduced diffusion of nutrients and oxygen within the hydrogel [8], or limited cell-scaffold interaction which could lead to anoikis [46]. Our results show that celastrol significantly protects encapsulated bone marrow-derived MSC viability and further work will be required to determine the mechanisms contributing to this observation.

In addition to increased cell viability, this study showed that preconditioning by celastrol also increases MSC paracrine function. This key feature for effective cell therapy has involved proangiogenic, anti-inflammatory, and antiapoptotic cytokines [17, 47]. In the case of ischemic diseases, proangiogenic paracrine activities contribute to wound healing by initiating cell proliferation, migration, and maturation for tissue revascularization [13, 48, 49]. Celastrol treatment was shown to significantly increase VEGFa and SDF-1*α* release, whereas the stimulated release of FGF-2 was also increased by celastrol; however, the difference did not reach statistical significance compared to the vehicle treatment group. It is important to mention however that this trend for FGF2 should not equate to a lack of functional significance considering that these experimental observations were made at a given time point, whereas in a translational situation, the combination of increased cell lifetime and paracrine stimulation over a sustained period of time may lead to significant benefits.

VEGFa, SDF-1*α*, and FGF2 factors measured in the present study are responsible for angiogenesis, proliferation, migration, expressions of adhesion protein, and extracellular matrix reconstruction [17, 18, 30, 50–52] and are involved in initiating tissue repair and reperfusion following ischemia [49, 53, 54]. The celastrol-stimulated increase in released factors observed in the present study may be partly due to the enhanced cell viability within the hydrogel. However, the higher level of VEGFa release (3.0-fold) compared to increase in cell viability (1.6-fold) supports enhanced production and/or release stimulated by celastrol and independently of cell number maintenance. Further studies are required to elucidate the important mechanism of enhanced paracrine factor production and release stimulated by celastrol.

Many studies have successfully improved the paracrine functions of MSC by exposing them to the sublethal environmental stress [29, 55–58] or by modifying or transfecting cell survival genes [30, 31]. Cell preconditioning with celastrol has the advantage to trigger the synthesis pathways of proangiogenic and cytoprotective growth factors mimicking the ischemic environment [33]. This might allow a rapid therapeutic effect when transplanted into ischemic tissue. The benefit of celastrol on the proangiogenic activity of encapsulated MSC was confirmed in the in vitro models herein. The observed benefits were greater in the HUVEC growth model probably due to the continuous stimulation of MSC in the coculture model, while in the wound healing model, the MSC-conditioned medium was harvested at a particular time point and conserved prior to its addition to media that was overlaid on the wounded HUVEC monolayer. Based on these promising in vitro results, the MSC-loaded gel was also tested in the rat subcutaneous model. An increase in neovessel density was observed in the periphery of the implant 7 days after injection of the hydrogel containing celastrol-treated MSC compared to the control (gel-only) condition. This said, statistical significance was not reached when comparing neovessel density of the celastrol-treated MSC compared to vehicle-treated MSC possibly due to the limited number of animals in each group and the single time point of observation at 7-day postimplantation. Moreover, the number of neovessels around the implanted hydrogel does not allow to conclude about their ability to reperfuse ischemic tissues.

To ensure optimal cell viability and release of proangiogenic factors and rapid vascularization of the implant in the target tissue, the hydrogel can also be created under the form of microbeads that ensure better diffusion of oxygen, nutrients, and secreted factors, as recently demonstrated by our team [59]. This format could also promote the formation of a vascularized network. Several methods have been developed to optimize cell survival and function in scaffolds, including scaffold modification with extracellular matrix proteins [21, 44, 60, 61] or addition of bioactive agents [41, 62]. Although interesting, these approaches could affect the mechanical and physicochemical properties of the scaffold [41]. The advantage of the present method is that it could be applied on any scaffold and possibly on a variety of cell types, as suggested by our previous work [34, 39]. Another advantage is the short duration of the cell preconditioning treatment is the possibility to completely wash out celastrol before cell encapsulation in the prehydrogel solution.

## 5. Conclusion

In this study, we demonstrated that simple and rapid pharmacological preconditioning of MSC with low doses of celastrol significantly enhances viability and paracrine function of cells encapsulated in an injectable thermosensitive hydrogel. This strategy could be applied using different kinds of scaffolds, cell types, conditioning molecules, and compound combinations. Our next step will be to demonstrate the benefit of cell preconditioning and encapsulation in cardiac and hind limb ischemia/reperfusion injury preclinical models. Altogether, combining cell encapsulation and pharmacological preconditioning is a promising strategy to enhance MSC survival, retention, and therapeutic function in order to improve outcomes of cell therapy and regenerative medicine applications.

## Figures and Tables

**Table 1:** Score of neovessel density in the peri-implant region after 7 days.

Score results			
Samples	Low response (+, ++)	High response (+++)	
Control	5/6 (83%)	1/6 (17%)	^∗^ *p* < 0.05
Vehicle-MSC	3/8 (37%)	5/8 (63%)	
Celastrol-MSC	1/8 (12%)	7/8 (88%)	

## Data Availability

The authors confirm that the data supporting the findings of this study are available within the article and its supplementary materials. If you need numerical data, Mr. Touani will be able to provide them to you.

## Supplementary Materials

Supplementary Materials Figure 1 S: schematic overview of hydrogel preparation. Figure 2 S: preliminary assay to determine the optimum celastrol concentration for cell preconditioning: MSC cultured in 24-well plates for 24 hours were treated with celastrol at various concentrations (0, 1 *μ*M, and 10 nM), coated with 500 *μ*L of blank hydrogel (3 mm thickness) and incubated with alpha MEM for 48 hours. (a) Viability of hMSC preconditioned with 10 nM (10^−8^) or 1 *μ*M (10^−6^) of celastrol and covered with a 3 mm hydrogel layer for 48 h. Mean ± SEM, *n* = 6, *N* = 2 (^∗^*p* < 0.05); (b) live/dead pictures (viable cells: green, dead cells: red), scale bar 200 *μ*m. Figure 3 S: (a) viability of rMSC preconditioned with 10 nM (10^−8^) or 1 *μ*M (10^−6^) of celastrol or vehicle (DMSO 0.1% *v*/*v*) and covered with a 3 mm hydrogel layer for 48 h. Mean ± SEM, *n* = 6, *N* = 2 (^∗^*p* < 0.05). (b) Live/dead pictures (viable cells: green, dead cells: red), scale bar 200 *μ*m. Figure 4 S: celastrol preconditioning increases the amount of angiogenic factors released by encapsulated hMSC. (A) VEGF-a (mean ± SEM, *n* ≥ 6, *N* = 4), (B) SDF-1*α* (mean ± SEM, *n* ≥ 12, *N* = 4), and (C) FGF-2 (mean ± SEM, *n* = 6, *N* = 3) concentrations in conditioned media of hydrogel encapsulated hMSC on day 4. Table 1S: raw data scores of neovessel density in the peri-implant region.

## References

[1] World Health Organization About cardiovascular diseases. http://www.who.int/cardiovascular_diseases/about_cvd/en/.

[2] World Health Organization Maladies cardiovasculaires. http://www.who.int/mediacentre/factsheets/fs317/fr/.

[3] Michaels A. D., Chatterjee K. (2002). Cardiology patient pages. Angioplasty versus bypass surgery for coronary artery disease. *Circulation*.

[4] Jeger R. V., Farah A., Ohlow M. A. (2018). Drug-coated balloons for small coronary artery disease (BASKET-SMALL 2): an open-label randomised non-inferiority trial. *The Lancet*.

[5] Palmerini T., Benedetto U., Biondi-Zoccai G. (2015). Long-term safety of drug-eluting and bare-metal stents: evidence from a comprehensive network meta-analysis. *Journal of the American College of Cardiology*.

[6] Levin S. R., Arinze N., Siracuse J. J. (2020). Lower extremity critical limb ischemia: a review of clinical features and management. *Trends in Cardiovascular Medicine*.

[7] Veenstra E. B., van der Laan M. J., Zeebregts C. J., de Heide E. J., Kater M., Bokkers R. P. H. (2020). A systematic review and meta-analysis of endovascular and surgical revascularization techniques in acute limb ischemia. *Journal of Vascular Surgery*.

[8] Russo V., Young S., Hamilton A., Amsden B. G., Flynn L. E. (2014). Mesenchymal stem cell delivery strategies to promote cardiac regeneration following ischemic injury. *Biomaterials*.

[9] Qadura M., Terenzi D. C., Verma S., al-Omran M., Hess D. A. (2018). Concise review: cell therapy for critical limb ischemia: an integrated review of preclinical and clinical studies. *Stem Cells*.

[10] Jeong H., Yim H. W., Park H. J. (2018). Mesenchymal stem cell therapy for ischemic heart disease: systematic review and meta-analysis. *International Journal of Stem Cells*.

[11] Trindade F., Leite-Moreira A., Ferreira-Martins J., Ferreira R., Falcão-Pires I., Vitorino R. (2017). Towards the standardization of stem cell therapy studies for ischemic heart diseases: bridging the gap between animal models and the clinical setting. *International Journal of Cardiology*.

[12] Sylakowski K., Bradshaw A., Wells A. (2020). Mesenchymal stem cell/multipotent stromal cell augmentation of wound healing: lessons from the physiology of matrix and hypoxia support. *The American Journal of Pathology*.

[13] Sears V., Ghosh G. (2020). Harnessing mesenchymal stem cell secretome: effect of extracellular matrices on proangiogenic signaling. *Biotechnology and Bioengineering*.

[14] Benoit E., O'donnell T. F., Patel A. N. (2013). Safety and efficacy of autologous cell therapy in critical limb ischemia: a systematic review. *Cell Transplantation*.

[15] Zhao L., Johnson T., Liu D. (2017). Therapeutic angiogenesis of adipose-derived stem cells for ischemic diseases. *Stem Cell Research & Therapy*.

[16] Li L., Chen X., Wang W. E., Zeng C. (2016). How to improve the survival of transplanted mesenchymal stem cell in ischemic heart?. *Stem Cells International*.

[17] Karpov A. A., Udalova D. V., Pliss M. G., Galagudza M. M. (2017). Can the outcomes of mesenchymal stem cell-based therapy for myocardial infarction be improved? Providing weapons and armour to cells. *Cell Proliferation*.

[18] Rehman J., Traktuev D., Li J. (2004). Secretion of angiogenic and antiapoptotic factors by human adipose stromal cells. *Circulation*.

[19] Tang Y. L., Zhao Q., Zhang Y. C. (2004). Autologous mesenchymal stem cell transplantation induce VEGF and neovascularization in ischemic myocardium. *Regulatory Peptides*.

[20] Roche E. T., Hastings C. L., Lewin S. A. (2014). Comparison of biomaterial delivery vehicles for improving acute retention of stem cells in the infarcted heart. *Biomaterials*.

[21] Karoubi G., Ormiston M. L., Stewart D. J., Courtman D. W. (2009). Single-cell hydrogel encapsulation for enhanced survival of human marrow stromal cells. *Biomaterials*.

[22] Young S. A. (2017). *Mechanically robust injectable hydrogel scaffolds for the intramuscular delivery of adipose-derived stem/stromal cells, [Ph.D. thesis]*.

[23] Mayfield A. E., Tilokee E. L., Latham N. (2014). The effect of encapsulation of cardiac stem cells within matrix-enriched hydrogel capsules on cell survival, post-ischemic cell retention and cardiac function. *Biomaterials*.

[24] Young S., Flynn L. E., Amsden B. G. (2015). A mechanically robust injectable hydrogel scaffold for adipose-derived stem cell delivery for the treatment of peripheral arterial disease. *Tissue Engineering Part A*.

[25] Chenite A., Chaput C., Wang D. (2000). Novel injectable neutral solutions of chitosan form biodegradable gels in situ. *Biomaterials*.

[26] Ceccaldi C., Assaad E., Hui E., Buccionyte M., Adoungotchodo A., Lerouge S. (2017). Optimization of injectable thermosensitive scaffolds with enhanced mechanical properties for cell therapy. *Macromolecular Bioscience*.

[27] Assaad E., Maire M., Lerouge S. (2015). Injectable thermosensitive chitosan hydrogels with controlled gelation kinetics and enhanced mechanical resistance. *Carbohydrate Polymers*.

[28] Uemura R., Xu M., Ahmad N., Ashraf M. (2006). Bone marrow stem cells prevent left ventricular remodeling of ischemic heart through paracrine signaling. *Circulation Research*.

[29] Hu X., Yu S. P., Fraser J. L. (2008). Transplantation of hypoxia-preconditioned mesenchymal stem cells improves infarcted heart function via enhanced survival of implanted cells and angiogenesis. *The Journal of Thoracic and Cardiovascular Surgery*.

[30] Zhang D., Fan G. C., Zhou X. (2008). Over-expression of CXCR4 on mesenchymal stem cells augments myoangiogenesis in the infarcted myocardium. *Journal of Molecular and Cellular Cardiology*.

[31] Huang B., Qian J., Ma J. (2014). Myocardial transfection of hypoxia-inducible factor-1*α* and co-transplantation of mesenchymal stem cells enhance cardiac repair in rats with experimental myocardial infarction. *Stem Cell Research & Therapy*.

[32] Noiseux N., Borie M., Desnoyers A. (2012). Preconditioning of stem cells by oxytocin to improve their therapeutic potential. *Endocrinology*.

[33] Der Sarkissian S., Le Huu A., Borie M., Hamet P., Noiseux N. Priming of stem cells with celastrol to enhance survival for cell therapy.

[34] Der Sarkissian S., Lévesque T., Noiseux N. (2017). Optimizing stem cells for cardiac repair: current status and new frontiers in regenerative cardiology. *World Journal of Stem Cells*.

[35] Gonzalez-Reyes A., Menaouar A., Yip D. (2015). Molecular mechanisms underlying oxytocin-induced cardiomyocyte protection from simulated ischemia-reperfusion. *Molecular and Cellular Endocrinology*.

[36] Zhao J., Young Y. K., Fradette J., Eliopoulos N. (2015). Melatonin pretreatment of human adipose tissue-derived mesenchymal stromal cells enhances their prosurvival and protective effects on human kidney cells. *American Journal of Physiology-Renal Physiology*.

[37] Venkatesha S. H., Dudics S., Astry B., Moudgil K. D. (2016). Control of autoimmune inflammation by celastrol, a natural triterpenoid. *Pathogens and Disease*.

[38] Venkatesha S. H., Moudgil K. D. (2016). Celastrol and its role in controlling chronic diseases. *Anti-inflammatory Nutraceuticals and Chronic Diseases*.

[39] Der Sarkissian S., Cailhier J. F., Borie M. (2014). Celastrol protects ischaemic myocardium through a heat shock response with up-regulation of haeme oxygenase-1. *British Journal of Pharmacology*.

[40] Noiseux N., Gnecchi M., Lopez-Ilasaca M. (2006). Mesenchymal stem cells overexpressing Akt dramatically repair infarcted myocardium and improve cardiac function despite infrequent cellular fusion or differentiation. *Molecular Therapy*.

[41] Alinejad Y., Adoungotchodo A., Hui E., Zehtabi F., Lerouge S. (2018). An injectable chitosan/chondroitin sulfate hydrogel with tunable mechanical properties for cell therapy/tissue engineering. *International Journal of Biological Macromolecules*.

[42] Elhami E., Dietz B., Xiang B. (2013). Assessment of three techniques for delivering stem cells to the heart using PET and MR imaging. *EJNMMI Research*.

[43] Robey T. E., Saiget M. K., Reinecke H., Murry C. E. (2008). Systems approaches to preventing transplanted cell death in cardiac repair. *Journal of Molecular and Cellular Cardiology*.

[44] Rustad K. C., Wong V. W., Sorkin M. (2012). Enhancement of mesenchymal stem cell angiogenic capacity and stemness by a biomimetic hydrogel scaffold. *Biomaterials*.

[45] Monette A., Ceccaldi C., Lerouge S., Lapointe R. (2016). Chitosan thermogels for local T lymphocyte delivery for cancer immunotherapy. *Biomaterials*.

[46] Zvibel I., Smets F., Soriano H. (2002). Anoikis: Roadblock to Cell Transplantation?. *Cell Transplantation*.

[47] Pham T. L.-B., Van Pham P. (2016). The effects of transplanted cells in stem cell therapy for myocardial ischemia. *Biomedical Research and Therapy*.

[48] Goodwin A. M. (2007). In vitro assays of angiogenesis for assessment of angiogenic and anti- angiogenic agents. *Microvascular Research*.

[49] Lu H., Wang F., Mei H., Wang S., Cheng L. (2018). Human adipose mesenchymal stem cells show more efficient angiogenesis promotion on endothelial colony-forming cells than umbilical cord and endometrium. *Stem Cells International*.

[50] Sadat S., Gehmert S., Song Y. H. (2007). The cardioprotective effect of mesenchymal stem cells is mediated by IGF-I and VEGF. *Biochemical and Biophysical Research Communications*.

[51] Murakami M., Nguyen L. T., Hatanaka K. (2011). FGF-dependent regulation of VEGF receptor 2 expression in mice. *The Journal of Clinical Investigation*.

[52] Srisakuldee W., Makazan Z., Nickel B. E. (2014). The FGF-2-triggered protection of cardiac subsarcolemmal mitochondria from calcium overload is mitochondrial connexin 43-dependent. *Cardiovascular Research*.

[53] Kokai L. E., Marra K., Rubin J. P. (2014). Adipose stem cells: biology and clinical applications for tissue repair and regeneration. *Translational Research*.

[54] Zhao W., Li J., Qiu X., Li S., Jin K. (2017). Delivery of stromal cell-derived factor 1*α* for in situ tissue regeneration. *Journal of Biological Engineering*.

[55] Rasmussen J. G., Frøbert O., Pilgaard L. (2011). Prolonged hypoxic culture and trypsinization increase the pro-angiogenic potential of human adipose tissue-derived stem cells. *Cytotherapy*.

[56] Hadjipanayi E., Schilling A. F. (2013). Hypoxia-based strategies for angiogenic induction: the dawn of a new era for ischemia therapy and tissue regeneration. *Organogenesis*.

[57] Tong C., Hao H., Xia L. (2016). Hypoxia pretreatment of bone marrow—derived mesenchymal stem cells seeded in a collagen-chitosan sponge scaffold promotes skin wound healing in diabetic rats with hindlimb ischemia. *Wound Repair and Regeneration*.

[58] Taylor C., Church J. E., Williams M. D. (2018). Hypoxic preconditioning of myoblasts implanted in a tissue engineering chamber significantly increases local angiogenesis via upregulation of myoblast vascular endothelial growth factor-a expression and downregulation of miRNA-1, miRNA-206 and angiopoieti. *Journal of Tissue Engineering and Regenerative Medicine*.

[59] Alinejad Y., Bitar C. M. E., Martinez Villegas K., Perignon S., Hoesli C. A., Lerouge S. (2020). Chitosan microbeads produced by one-step scalable stirred emulsification: a promising process for cell therapy applications. *ACS Biomaterials Science & Engineering*.

[60] Liu Z., Wang H., Wang Y. (2012). The influence of chitosan hydrogel on stem cell engraftment, survival and homing in the ischemic myocardial microenvironment. *Biomaterials*.

[61] Singh D. J. (2017). Investigation of chitosan-based hydrogels as a cell delivery platform for adipose-derived stem/stromal cell transplantation to promote angiogenesis in ischemic tissues. *Anatomy and Cell Biology*.

[62] Hastings C. L., Kelly H. M., Murphy M. J., Barry F. P., O'Brien F. J., Duffy G. P. (2012). Development of a thermoresponsive chitosan gel combined with human mesenchymal stem cells and desferrioxamine as a multimodal pro-angiogenic therapeutic for the treatment of critical limb ischaemia. *Journal of Controlled Release*.

