# Supplementary material for: Pharmacological Preconditioning Improves the Viability and Proangiogenic Paracrine Function of Hydrogel-Encapsulated Mesenchymal Stromal Cells
Source: Stem Cells Int. 2021 Jul 28;2021:6663467. doi: 10.1155/2021/6663467 (PMC8342149; doi:10.1155/2021/6663467)
Supplement: Supplementary Materials — Figure 1 S: schematic overview of hydrogel preparation. Figure 2 S: preliminary assay to determine the optimum celastrol concentration for cell preconditioning: MSC cultured in 24-well plates for 24 hours were treated with celastrol at various concentrations (0, 1 μM, and 10 nM), coated with 500 μL of blank hydrogel (3 mm thickness) and incubated with alpha MEM for 48 hours. (a) Viability of hMSC preconditioned with 10 nM (10−8) or 1 μM (10−6) of celastrol and covered with a 3 mm hydrogel layer for 48 h. Mean ± SEM, n = 6, N = 2 (∗p < 0.05); (b) live/dead pictures (viable cells: green, dead cells: red), scale bar 200 μm. Figure 3 S: (a) viability of rMSC preconditioned with 10 nM (10−8) or 1 μM (10−6) of celastrol or vehicle (DMSO 0.1% v/v) and covered with a 3 mm hydrogel layer for 48 h. Mean ± SEM, n = 6, N = 2 (∗p < 0.05). (b) Live/dead pictures (viable cells: green, dead cells: red), scale bar 200 μm. Figure 4 S: celastrol preconditioning increases the amount of angiogenic factors released by encapsulated hMSC. (A) VEGF-a (mean ± SEM, n ≥ 6, N = 4), (B) SDF-1α (mean ± SEM, n ≥ 12, N = 4), and (C) FGF-2 (mean ± SEM, n = 6, N = 3) concentrations in conditioned media of hydrogel encapsulated hMSC on day 4. Table 1S: raw data scores of neovessel density in the peri-implant region. [file 6663467.f1.docx]

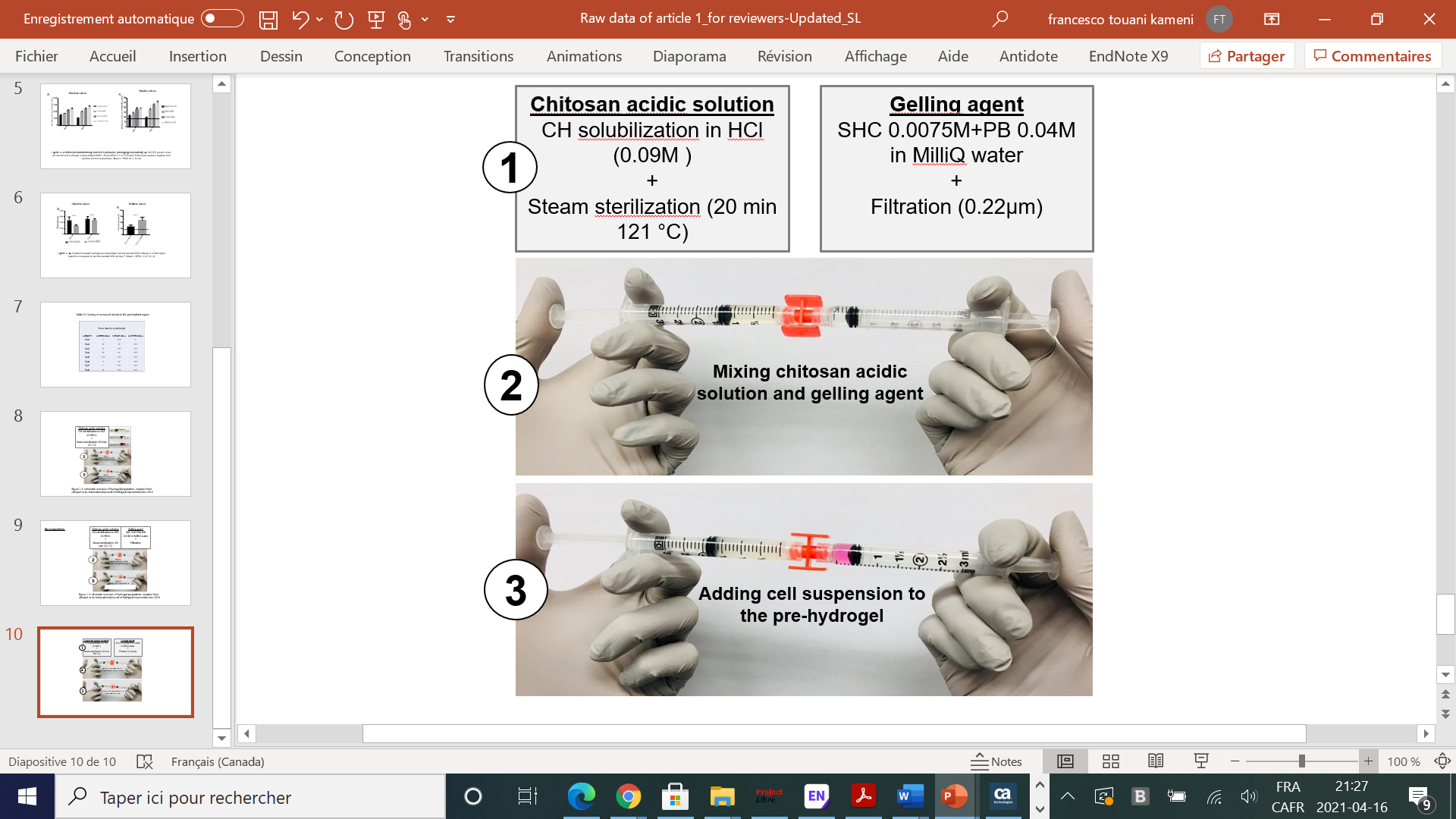


Figure 1 S : schematic overview of hydrogel preparation. Adapted from Alinejad et al., International journal of biological macromolecules. 2018


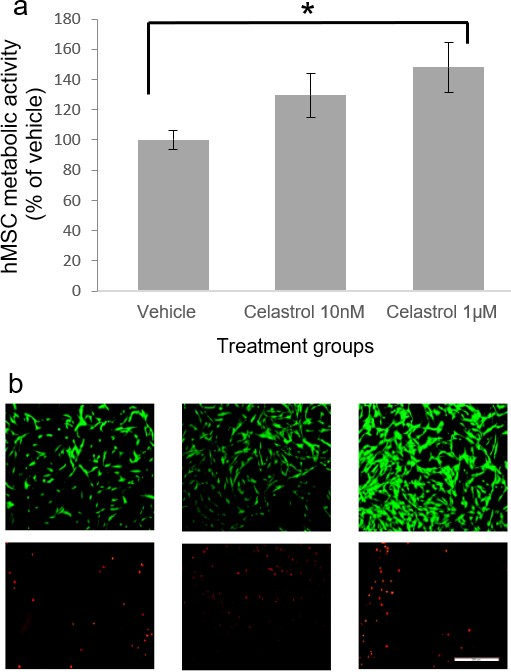


Vehicle Celastrol (10nM) Celastrol (1µM)

**Figure 2 S**: Preliminary assay to determine the optimum celastrol concentration for cell preconditioning: MSC cultured in 24-well plates for 24 hours were treated with celastrol at various concentrations (0, 1µM,10 nM), coated with 500 µL of blank hydrogel (3 mm thickness) and incubated with alpha MEM for 48 hours. a)Viability of hMSC preconditioned with 10 nM (10 -8) or 1 µM (10 -6) of celastrol and covered with a 3 mm hydrogel layer for 48 h. Mean ±SEM n=6, N=2 (*p < 0.05); b) live dead pictures (viable cells: green, dead cells: red), scale bar 200µm.


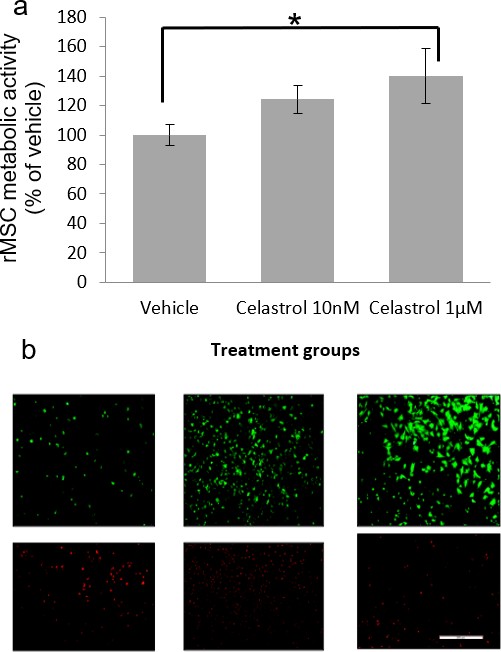


Vehicle Celastrol (10nM) Celastrol (1µM)

**Figure 3 S**. a) Viability of rMSC preconditioned with 10 nM (10 -8) or 1 µM (10 -6) of Celastrol or vehicle (DMSO 0.1% v/v) and covered with a 3 mm hydrogel layer for 48 h. Mean ±SEM n=6, N=2 (*p < 0.05). b) Live dead pictures (viable cells: green, dead cells: red), scale bar 200 µm.


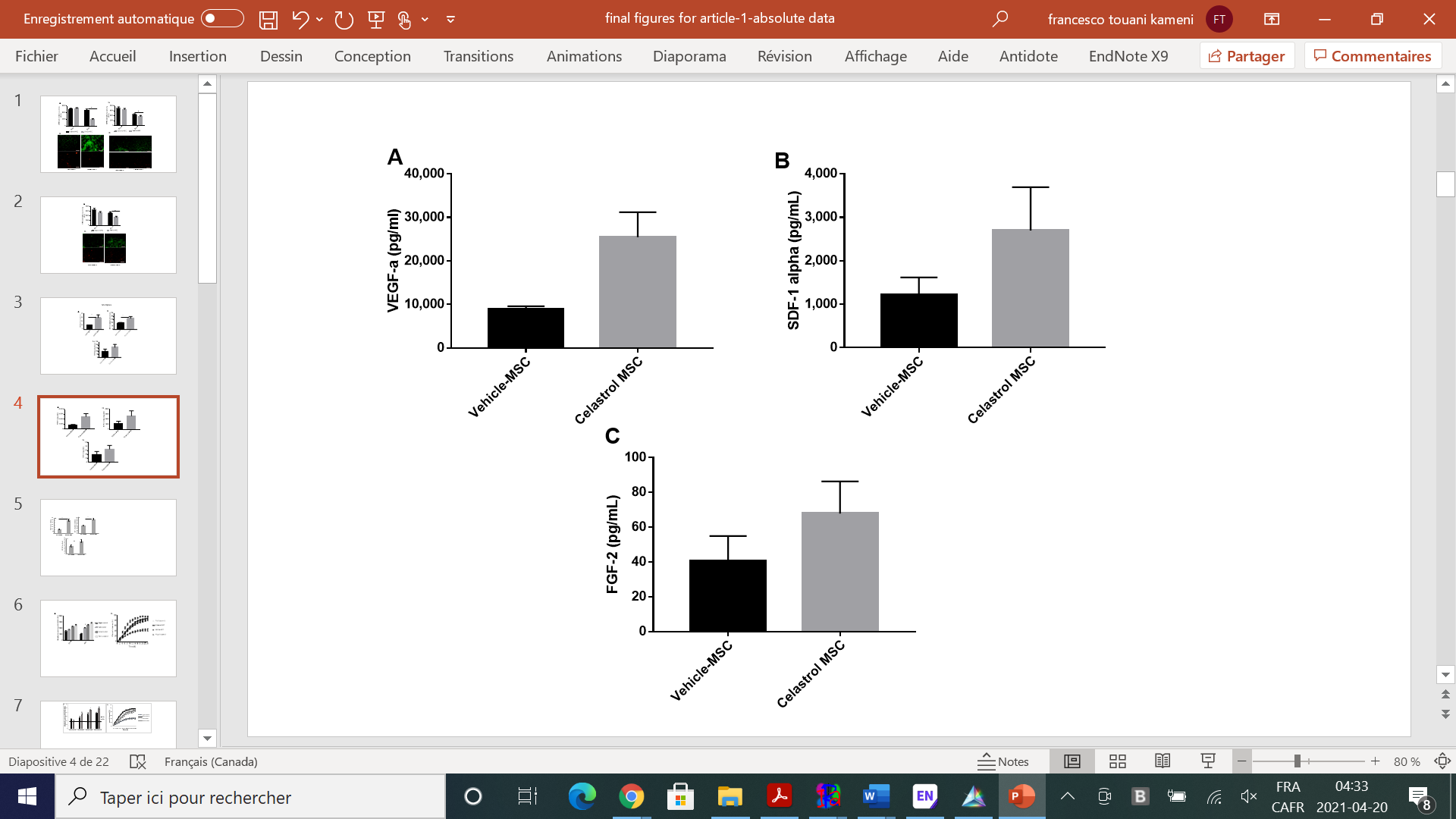


**Figure 4S: Celastrol preconditioning increases the amount of angiogenic factors released by encapsulated hMSC. A**) VEGF-a (Mean ±SEM n≥6, N=4), **B**) SDF-1α (Mean ±SEM n≥12, N=4) and **C**) FGF-2 (Mean ±SEM n=6, N=3) concentrations in conditioned media of hydrogel encapsulated hMSC on day 4

| **Samples** | **Control-MSC** | **Vehicle-MSC** | **Celastrol-MSC** |
| --- | --- | --- | --- |
| Rat1 | / | +++ | + |
| Rat2 | ++ | ++ | +++ |
| Rat3 | ++ | +++ | +++ |
| Rat4 | ++ | ++ | +++ |
| Rat5 | +++ | +++ | +++ |
| Rat6 | + | ++ | +++ |
| Rat7 | / | +++ | +++ |
| Rat8 | ++ | +++ | +++ |

**Table 1S:** Raw data scores of neovessel density in the peri implant region
